# Aligned electrospun poly(l-lactide) nanofibers facilitate wound healing by inhibiting macrophage M1 polarization via the JAK-STAT and NF-κB pathways

**DOI:** 10.1186/s12951-022-01549-9

**Published:** 2022-07-26

**Authors:** Jian Xie, Xiaowei Wu, Shang Zheng, Kaili Lin, Jiansheng Su

**Affiliations:** 1 Department of Prosthodontics, Stomatological Hospital and Dental School of Tongji University, Shanghai Engineering Research Center of Tooth Restoration and Regeneration, Shanghai, 200072 China; 2 Department of Orthodontics, Shanghai Ninth People’s Hospital, College of Stomatology, Shanghai Key Laboratory of Stomatology & Shanghai Research Institute of Stomatology, Shanghai Jiao Tong University School of Medicine, National Clinical Research Center for Oral Diseases, Shanghai, 200125 China; 3 Department of Oral & Cranio-Maxillofacial Surgery, Shanghai Ninth People’s Hospital, College of Stomatology, Shanghai Key Laboratory of Stomatology, Shanghai Research Institute of Stomatology, Shanghai Jiao Tong University School of Medicine, National Clinical Research Center for Oral Diseases, Shanghai, 200125 China

**Keywords:** Aligned nanofibers, Macrophage polarization, Inflammation, Wound healing, JAK-STAT, NF-κB

## Abstract

Delayed wound healing remains a challenge, and macrophages play an important role in the inflammatory process of wound healing. Morphological changes in macrophages can affect their phenotype, but little is known about the underlying mechanism. Aligned electrospun nanofibers have natural advantages in modulating cell morphology. Therefore, the current study constructed aligned electrospun nanofibers that could transform macrophages into elongated shapes. Our results demonstrated that aligned nanofibers without exogenous cytokines could downregulate the proinflammatory M1 phenotype and upregulate the prohealing M2 phenotype in an inflammatory environment. Importantly, our study revealed that aligned electrospun nanofibers could inhibit macrophage M1 polarization via the JAK-STAT and NF-κB pathways. Furthermore, the conditioned medium from macrophages cultured on aligned nanofibers could encourage fibroblast migration, proliferation and collagen secretion. In vivo, aligned nanofibers alleviated the inflammatory microenvironment, promoted angiogenesis and accelerated wound healing in mouse skin defects by modulating macrophage phenotypes. Collectively, aligned electrospun nanofibers can influence macrophage polarization via the JAK-STAT and NF-κB pathways and attenuate the local inflammatory response in skin wounds. This study provides a potential strategy to modulate macrophage polarization and promote wound healing by controlling the topology of biomaterials and offers a new perspective for the application of nanotechnology in wound healing.

**Supplementary Information:**

The online version contains supplementary material available at 10.1186/s12951-022-01549-9.

## Introduction

Cutaneous wound healing is a complex process of skin repair and regeneration following infection or mechanical trauma and involves four phases: hemostasis, inflammation, proliferation and remodeling [1]. Favorable wound healing requires numerous extracellular components and the interactions of various cell types, such as immune cells, fibroblasts, and endothelial cells [2, 3]. Among these, macrophages, which are indispensable cellular members of intrinsic immunity, are key coordinators of normal wound healing and tissue regeneration, especially during the inflammatory phase of healing [4, 5].

Macrophages can exhibit a range of different activation phenotypes in response to different microenvironmental or exogenous stimuli. When macrophages are exposed to invading intracellular pathogens or bacteria, they usually polarized to the M1 phenotype (the classic activation phenotype) [6]. M1 macrophages typically appear in proinflammatory environments governed by Toll-like receptor (TLR) or interferon (IFN) signaling. These cells are characterized by high expression of proinflammatory factors such as tumor necrosis factor-α (TNF-α), interleukin-1β (IL-1β), and inducible nitric oxide synthase (iNOS) [6]. M2 macrophages, which are the alternative activation phenotype, are present in a Th2 response-dominated environment and are induced by interleukin-4 (IL-4) or interleukin-13 (IL-13) [2]. M2 macrophages express high levels of Arginase-1 (Arg-1), which catalyzes the production of ornithine. Ornithine serves as the direct substrate for the cellular production of polyamines and is required for M2 macrophages to perform functions such as collagen synthesis, proliferation, and tissue remodeling [6, 7].

Classically activated (M1) macrophages secrete proinflammatory factors and exhibit enhanced microbicidal activity and high antigen-presenting capacity [8]. These features are facilitated by IFN-γ-mediated Janus kinase/signal transduction and activator of transcription (JAK-STAT) signaling. STAT1 is an important mediator of M1 macrophage polarization, and its activity is critical for M1 polarization [9]. Another key mechanism of M1 macrophage polarization is the nuclear factor-κB (NF-κB) signaling pathway. TLR activation on the membranes of macrophages initiates downstream cascades that activate the NF-κB pathway and promote the subsequent release of proinflammatory mediators [10].

The regression of inflammation is necessary for tissue regeneration, which involves a shift from the proinflammatory phenotype to the anti-inflammatory phenotype of macrophages. Studies have shown that cytoskeletal changes in macrophages can influence their phenotypes. M2 macrophages show an elongated shape compared with M1 cells [11]. Directly modulating the shape of macrophages to an elongated state by micropatterning methods in the absence of exogenous cytokines can promote M2 polarization and reduce the synthesis of proinflammatory cytokines [11]. Therefore, altering the cytoskeletal morphology of macrophages might be a promising strategy to modulate their phenotype.

Currently, with the development of nanotechnology, researchers have increasingly realized the importance of topological signals on cell morphology. Therefore, fabrication techniques such as laser structuring techniques and photolithography have been used to prepare biomaterials with micro- or nanopatterns that can modulate the morphology of cells and direct their fate [12, 13]. However, the widespread application of these technologies inevitably faces limitations such as high manufacturing expenses and complex processes.

In recent years, electrospinning has also been extensively used to modulate cell morphology due to its simplicity, convenience and affordability, and has been widely applied in wound healing [14, 15] and tissue regeneration [16–18]. Our previous work verified that aligned electrospun membranes could induce long spindle shapes rather than conventional polygons in rat bone marrow-derived macrophages (BMSCs) and promote their osteogenic differentiation [19]. Another study demonstrated the ability of aligned fibers to induce an elongated morphology and a prohealing phenotype of macrophages in a noninflammatory state [20]. Moreover, oriented microfibrils (average diameter 27.1 ± 3.9 μm) could contribute to the recruitment of macrophages and their subsequent transition to an anti-inflammatory phenotype [21]. These works confirmed that aligned electrospun fibers might have a regulatory effect on the macrophage phenotype. However, the effect of aligned nanofibers on macrophages in an inflammatory environment and the underlying mechanism are not yet known.

Poly (l-lactic acid) (PLLA) is a commonly used biodegradable polymer material. Its degradation product is lactic acid, which is also a byproduct of the normal metabolic process; therefore, PLLA has excellent biocompatibility [22]. More importantly, previous studies have shown that a proper amount of lactic acid could facilitate the lactylation of the promoter of Arg-1, an important enzyme in M2 polarization, which in turn promotes Arg-1 expression [23].

**Scheme 1:**
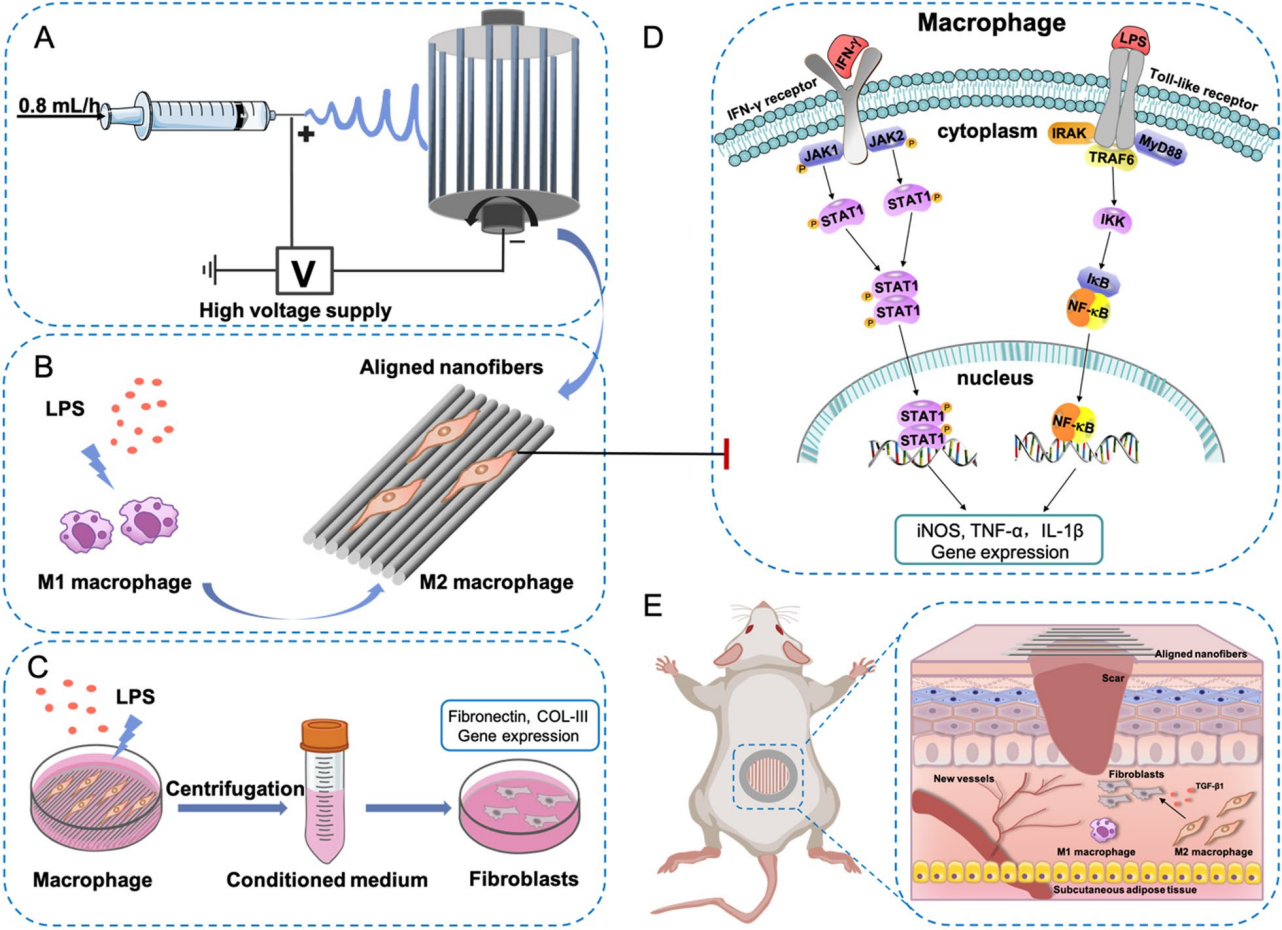
The fabrication of aligned electrospun PLLA nanofibers and their use in wound healing. **A** Schematic diagram showing the preparation of aligned electrospun nanofibers. **B** Aligned nanofibers promote M2 macrophage polarization. **C** The conditioned medium of macrophages cultured on aligned nanofibers promoted collagen secretion by fibroblasts. **D** Aligned nanofibers inhibit macrophage M1 polarization via the JAK-STAT and NF-κB signaling pathways. **E** Aligned nanofibers facilitate skin wound healing in mice

In this study, we aimed to elucidate the effects of aligned PLLA electrospun nanofibers on macrophage polarization in an LPS-induced inflammatory environment and preliminarily explore the molecular mechanism. The current study first revealed that aligned nanofibers could inhibit the lipopolysaccharide (LPS)-induced M1 macrophage phenotype via the JAK-STAT and NF-κB signaling pathways. The aligned nanofiber membranes also promoted skin wound healing in mice (Scheme 1). This finding suggests that simply changing the surface morphology of electrospun nanofibers can reverse the inflammatory environment and macrophage polarization without the addition of exogenous bioactive components, which provides a new strategy for the use of biomaterials in macrophage polarization and wound healing.

## Materials and methods

### Materials and reagents

PLLA with an average MW of 138,000 was purchased from Daigang Biomaterial Co. Ltd. (Jinan, China). PrimeScript RT Master Mix and FastStart Universal SYBR Green Master were purchased from Takara (Japan). Primary antibodies of β-actin (4970) and IL-1β (31,202) was purchased from CST Inc (USA). Primary antibodies of Arg-1 (DF6657), iNOS (AF0199), NF-κB p65 (AF5006), phospho-NF-κB p65 (AF2006), JAK-1 (AF5012), phospho-JAK-1 (AF2012) JAK-2 (AF6002), phospho-JAK-2 (AF3024), STAT1 (AF6300), phospho-STAT1 (AF3300), STAT3 (AF6294), phospho-STAT3 (AF3293) and TGF-β1 (AF1027) were purchased from Affinity BioScience LTD (China). Primary antibodies of CD206 (ab64693), CD86 (ab220188), CD31(ab28364) and Goat Anti-Rabbit IgG H&L (Alexa Fluro594, ab150080) were purchased from Abcam Plc.(USA). Anti-CD86/APC (bs-1035R-APC), Anti-macrophage mannose receptor 1/PE (bs-23178R-PE), Anti-F4/80/FITC (bs-11182R-FITC) and Goat Anti-Rabbit IgG H&L (bs-0295G) were purchased from Bioss Inc. (China).

### Fabrication and characterization of electrospun fibers

Electrospun fibers with different structures (aligned and random nanofibers) were fabricated according to our previous study [19]. Briefly, a 20% w/v solution of PLLA was first prepared. Then, the fibers were prepared by electrospinning (18 kV voltage, 0.8 mL/h flow rate, 18 cm collection distance) with an ordinary roller collector (30 rpm) to prepare random fibers and a cage-shaped roller collector (800 rpm) to prepare aligned fibers. The random electrospun nanofibers were named R20, while the aligned fibers were named A20. Then, the electrospun nanofiber membranes were sprayed with gold foil and observed by scanning electron microscopy (SEM). The diameters of both groups of electrospun nanofibers were calculated by ImageJ software. In addition, the mechanical properties of A20 and R20 (n = 3 for each group) were examined by a mechanical test machine (HengYi, China), and the water contact angles (n = 3 for each group) were measured by the sessile-drop technique (Sunzern, China).

### Cell culture

The electrospun membranes were sterilized according to our previous study [19]. RAW264.7 cells were seeded onto electrospun membranes and cultured with high-glucose DMEM (Gibco, USA) containing 10% fetal bovine serum (FBS, Gibco, USA) and 1% penicillin/streptomycin (HyClone, USA) for 3 days to allow for full morphological extension. The incubator was maintained at 37 ℃ with 95% humidity and 5% CO_2_. Cells cultured on the surface of the well served as the control group, which was named Con and received the same treatment. Then, 1 µg/mL LPS (Sigma, USA) was added to induce RAW264.7 cells to polarize to the M1 phenotype. 24 h later, the cells were collected for the following experiments.

### Flow cytometry

After 24 h of induction, RAW264.7 cells were collected, blocked for 1 h with 5% bull serum albumin (BSA, Beyotime, China) and incubated with antibodies (CD86-APC as a marker of the M1 phenotype and CD206-PE as a marker of the M2 phenotype) for 1 h to analyze macrophage polarization. Labeling was quantified with a BD FACS Verse flow cytometer (USA).

### Phalloidin fluorescent staining

To observe the morphologies of RAW264.7 cells on different electrospun membranes, cells were seeded on the samples and cultured for 3 days. The cells were fixed with 4% paraformaldehyde (PFA, Beyotime, China), followed by permeabilization with 0.5% Triton X-100 (Beyotime, China). Then, the cells were blocked with 1% BSA for 1 h, after which DAPI and FITC-labeled phalloidin (Sigma, USA) were used to label the nucleus and cytoskeleton, respectively. Finally, a laser confocal microscope (Nikon, Japan) was used to observe the images.

### Quantitative real-time polymerase chain reaction (qPCR)

After 24 h of induction with LPS, total RNA was isolated, and reverse transcription was performed to prepare complementary DNA. qPCR was then performed on a Light Cycler^®^ 96 Real-Time PCR System (Roche, Switzerland) with Fast Start Universal SYBR Green Master Mix (Takara RR820A, Japan). The expression of Arg-1, IL-4, IL-10, TGF-β, IL-1β, TNF-α and iNOS was examined, and GAPDH was used as a housekeeping gene. The calculation to normalize expression was performed based on the difference between the threshold values of the target gene and GAPDH. The primer sequences are shown in Additional file 1: Table S1.

### ELISA

Macrophage-conditioned medium was collected, and the levels of IL-4 were measured by an ELISA kit (EK0405, Boster, China) according to the manufacturer’s instructions.

### Western blotting

After 24 h of induction with LPS, RAW264.7 cells were lysed in RIPA reagent (Beyotime, China). A BCA kit (Beyotime, China) was used to measure the total protein concentration. Then, the proteins were separated by gel electrophoresis and transferred to NC membranes. The membranes were blocked for 1 h with 5% skimmed milk powder and incubated with primary antibodies against Arg-1, iNOS and IL-1β overnight at 4 °C. Then, the membranes were incubated with the secondary antibody for 1 h, and the expression of target proteins was visualized with an enhanced chemiluminescence detection system (Tanon V8, China).

### Immunofluorescence staining

After 24 h of induction with LPS, RAW264.7 cells were fixed with 4% PFA and permeabilized with 0.5% Triton X-100. Then, the cells were blocked with 1% BSA for 1 h, after which the primary antibodies (CD86, CD206 and NF-κB) were added and incubated overnight at 4 °C. The samples were rinsed with phosphate buffer saline (PBS, HyClone, USA) a few times and incubated with secondary antibodies for 1 h at room temperature. Then, DAPI and FITC-labeled phalloidin were used to label the nucleus and cytoskeleton, respectively. Finally, laser confocal microscopy was used to observe the images.

### RNA sequencing (RNA-seq)

Cell samples (n = 3 for each group) were obtained as described above and stored in RNAiso buffer, and the samples were analyzed by Personalbio Co., Ltd. Differential gene expression was analyzed by gene ontology (GO) and Kyoto Encyclopedia of Genes and Genomes (KEGG) to filter the related targets. Further validation of these results was performed by Western blotting.

### Conditioned medium of macrophages

After 24 h of induction with LPS, the supernatant of RAW264.7 cells was collected and mixed with DMEM at a ratio of 1:2, and this conditioned medium was named Con-CM, A20-CM, and R20-CM.

L929 fibroblasts and mouse arterial endothelial cells (MAECs) were seeded in 24-well plates and cultured with normal DMEM. Then, the medium was replaced with conditioned medium after 24 h of culture. Cell proliferation was examined by CCK-8 assays on the 1st, 4th and 7th days.

L929 fibroblasts/MAECs were seeded in 24-well plates and cultured with normal DMEM. After the cells had attached, a scratch was made using a pipette tip in the middle of the cells, and the medium was replaced with different conditioned medium. After 24 h, the cells were stained with DAPI. Photographs were taken by an inverted fluorescence microscope.

L929 cells/MAECs were seeded in 6-well plates and incubated with conditioned medium 24 h later. After the cells were cultured for 3 days, total RNA was isolated, and qPCR was performed to measure the expression of fibronectin, collagen-III (COL-III) and COL-I in L929 fibroblasts. For MAECs, the expression of kinase insert domain receptor (KDR), endothelial nitric oxide synthase (eNOS) and basic fibroblast growth factor (bFGF) was examined. Immunofluorescence staining of fibronectin in L929 cells was performed according to the protocols described above.

### Animal experiments

Eight-week-old male C57 mice were used for animal experiments (n = 5 for each group). All animal experiments were approved by the Animal Protection and Use Committee of Tongji University (Shanghai, China). All mice were subjected to isoflurane inhalation anesthesia, the dorsal hair was removed, skin defects with a diameter of 10 mm were prepared, and silicone rings were sutured around the defects. Aligned and random electrospun membranes were cut into circular patches with diameters of 10 mm, sterilized with 75% alcohol overnight and rinsed with PBS 3 times in advance. The patches were pasted on the surface of the skin defect. The defect group without electrospun membrane placement was used as the control group (Con). Skin defect healing in the mice was observed and photographed at various time points. The silicone rings were checked daily, and those that fell off were promptly replaced. The mice were sacrificed under isoflurane inhalation anesthesia on days 7 and 14, and 1 × 1 cm^2^ skin was excised and fixed in 4% PFA.

### Histological experiments

The collected samples were dehydrated and embedded in paraffin wax. The samples were cut into 5-µm-thick sections using a slicer (Leica, Germany). H&E and Masson staining were performed according to the instructions. Immunofluorescence staining for CD86 and CD206 was performed on tissue sections on days 7 and 14 to measure the polarization of macrophages in the samples. Xylene was used to dewax the paraffin sections, and gradient alcohol was used for rehydration. Then, antigen repair was performed with protease K and trypsin at 37 ℃, and then the sections were treated with H_2_O_2_ for 10 min and blocked with BSA for 1 h. The sections were incubated with primary antibodies at 4 °C overnight, and a secondary antibody was subsequently applied. TGF-β1, CD31 and iNOS immunofluorescence staining on days 7 and 14 was performed using the same procedure.

### Statistical analysis

The numerical data are expressed as the mean ± standard deviation. Statistical analysis of all experimental data was completed by one-way analysis of variance (ANOVA) and t tests. *p < 0.05 was considered statistically significant.

## Results and discussion

### Characterization of electrospun membranes

**Fig. 1:**
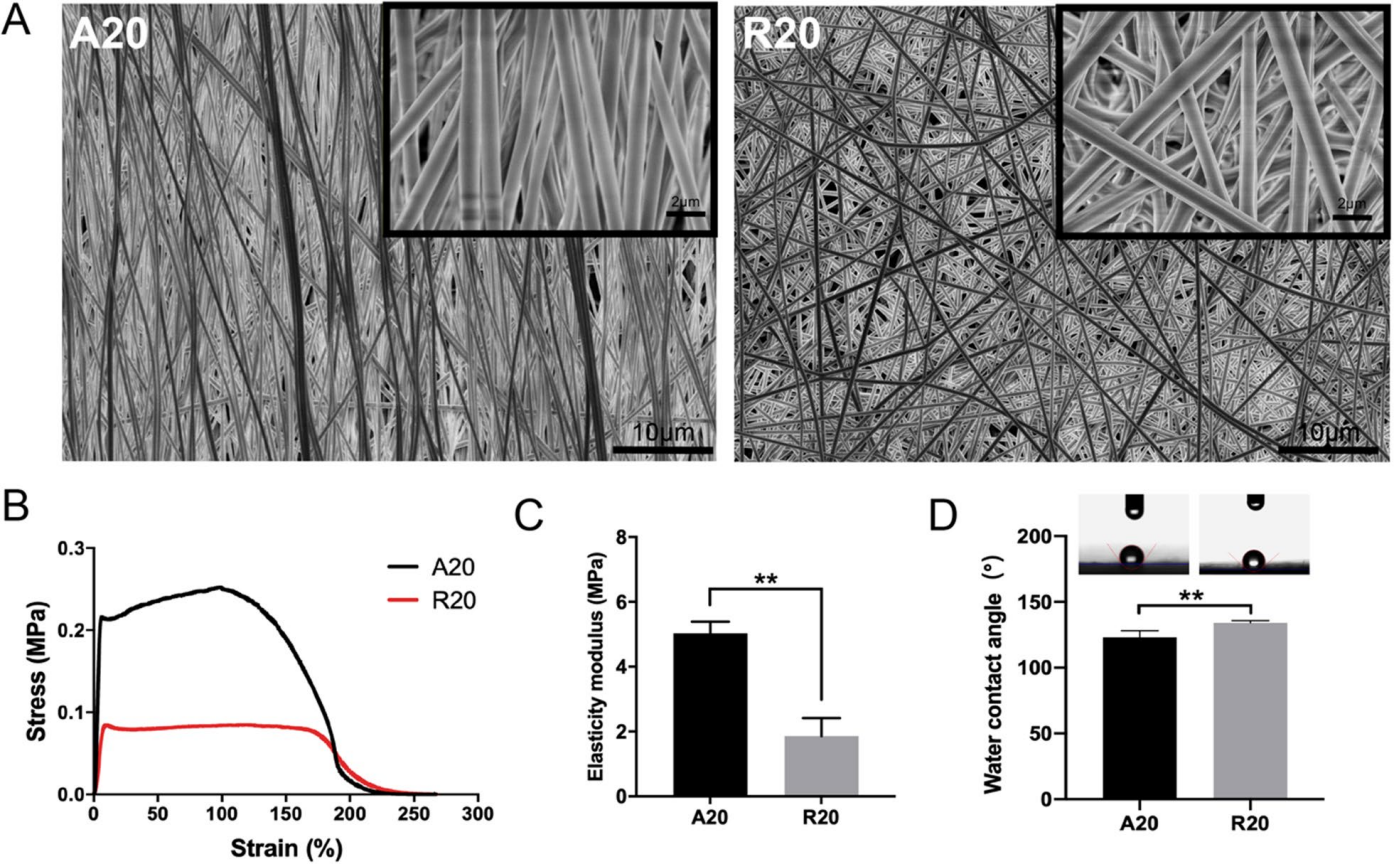
Characterization of electrospun membranes. **A** SEM images, **B** stress–strain curves, **C** elasticity modulus and **D** water contact angles of the aligned and random electrospun membranes. (**p < 0.01, n = 3)

The morphologies of aligned and random membranes were observed by SEM, as shown in Fig. 1A. The nanofibers in both groups presented smooth and continuous morphology, and the arrangement direction of the fibers in the A20 group was consistent, while that in the R20 group was disorderly. The diameters in both groups were calculated by ImageJ software. The mean diameter in the A20 group was 758 ± 102 nm, and the mean diameter in the R20 group was 730 ± 94 nm. There was no significant difference between the two groups (Additional file 1: Fig. S1). In order to investigate the mechanical properties and hydrophilicity of electrospun fiber membranes, and assess the feasibility of their later application in wound healing, mechanical properties and water contact angle were examined. Compared with the R20 group, the A20 group exhibited higher tensile strength (Fig. 1B) and elastic modulus (Fig. 1C). The water contact angle of the A20 group was significantly lower than that of the R20 group (Fig. 1D).

Previous studies have shown that the topology of biomaterials has a significant impact on subsequent cellular behaviors [24, 25]. Therefore, we sought to modulate the cytoskeletal structure of macrophages using electrospun fibrous membranes with different alignments and investigate their effects on macrophage polarization. It is generally believed that nanoscale biological scaffolds exhibit more advantages for tissue healing than micron-sized scaffolds due to their larger surface areas for the formation of more integrin adhesion sites [26]. After parameter optimization, highly aligned nanofibers were obtained. Significantly higher tensile strength and moduli of elasticity were observed in the A20 group than in the random group, which made these fibers more suitable for wound healing because they might provide more appropriate mechanical strength during tissue regeneration and reduce wound scar healing. Previous studies by Ma et al. [27] were consistent with our results, and the mechanical improvement was ascribed to the anisotropy of the aligned nanofibers. In addition, aligned nanofibers exhibited lower water contact angles than random nanofibers, indicating better hydrophilicity, which was reported to be beneficial for subsequent cell adhesion and other biological behaviors [28, 29].

### Aligned nanofibers inhibited the LPS-induced M1 macrophage phenotype

**Fig. 2:**
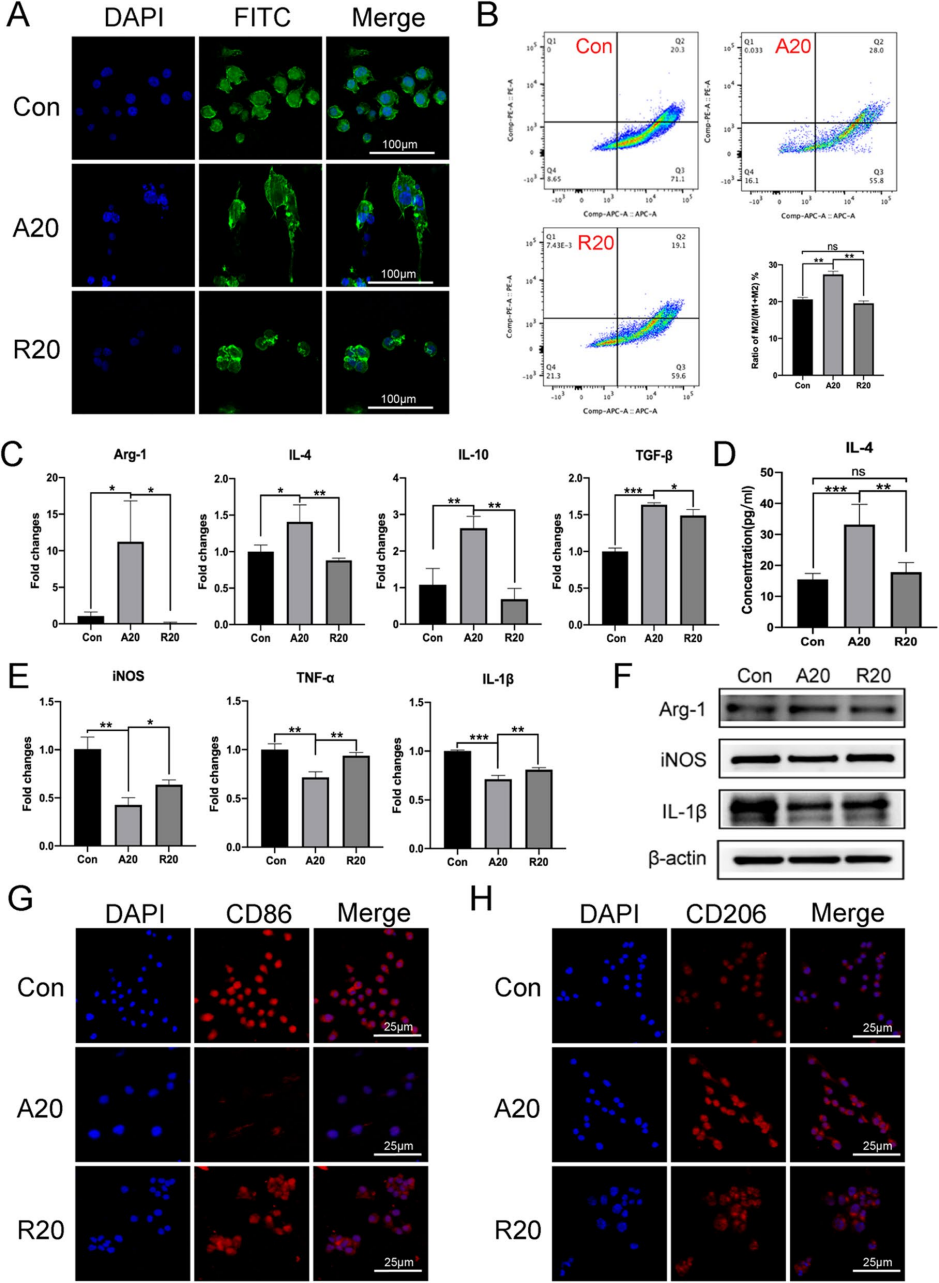
The effect of aligned nanofibers on macrophage polarization. **A** Phalloidin staining of macrophages on electrospun membranes. Actin is stained green, and the nucleus is stained blue. **B** Flow cytometric analysis of macrophages. **C** qPCR analysis of M2 polarization-related genes. **D** ELISA analysis of IL-4 secretion. **E** qPCR analysis of M1 polarization-related genes (*p < 0.05, **p < 0.01, ***p < 0.001, n = 3). **F** Western blot analysis of macrophage polarization-related proteins. **G** and **H** Immunofluorescence analysis of CD86 and CD206 expression in macrophages. CD86 and CD206 are stained red, and the nucleus is stained blue

To investigate the effects of aligned nanofibers on macrophage polarization, phalloidin staining was performed to observe the morphology of macrophages cultured on aligned and random nanofibers. The results showed that macrophages in the A20 group exhibited an elongated shape that extended along the fibers, while the macrophages in the other groups remained spherical (Fig. 2A). Flow cytometry showed that the proportion of M2 macrophages in the A20 group was the highest compared with that in the other two groups (Fig. 2B). Then, qPCR was performed to evaluate the expression of macrophage polarization-related genes. The A20 group had significantly upregulated expression of the M2 phenotype-related genes Arg-1, IL-4, IL-10 and TGF-β (Fig. 2C) and downregulated expression of the M1 phenotype-related genes TNF-α, IL-1β and iNOS (Fig. 2E). The ELISA results verified that the secretion of IL-4 in the A20 group was significantly higher than that in the Con and R20 groups, which was consistent with previous results (Fig. 2D). Western blotting images are shown in Fig. 2F, and the results revealed that Arg-1 expression was upregulated, while iNOS and IL-1β levels were downregulated in the A20 group. Immunofluorescence staining of CD86 and CD206 showed inhibition of M1 macrophages and promotion of M2 macrophages in the A20 group (Fig. 2G and H).

According to previous studies, the M2 macrophage phenotype usually exhibits a long spindle shape [30]. Similarly, induing macrophages to form spindle shapes with biomaterials promoted M2 polarization in macrophages [11]. It was reported that aligned electrospun nanofibers had natural advantages that prompted cells to become fusiform [31]. Thus, it might be reasonable that aligned electrospun fibers could influence the immunomodulatory functions of cells. For example, aligned electrospun microfibers allowed adipose-derived mesenchymal stem cells (ASCs) to synthesize more immunomodulatory-related factors than cells cultured on random fibers, and their conditioned medium enhanced the M2 polarization of macrophages [32]. The results of Jia et al. [20] also demonstrated that aligned nanofibers facilitated the prohealing phenotype in bone marrow-derived macrophage (BMDMs) and that aligned nanofiber-constructed nerve-guided conduits could promote peripheral nerve regeneration. However, the effect of aligned electrospun nanofibers on macrophage polarization in an inflammatory environment and the molecular mechanism remain unclear. We hypothesized that aligned nanofibers could inhibit M1 phenotype macrophage polarization under the induction of LPS. The phalloidin fluorescence images showed the morphological changes in macrophages cultured on aligned electrospun membranes. Subsequent flow cytometry, which is regarded as the gold standard, verified that the A20 group had decreased proportions of M1 macrophages and increased proportions of M2 macrophages. This finding indicated that aligned nanofibers could alter the M2/M1 ratio in an inflammatory microenvironment. Immunofluorescence staining of CD86 and CD206 also added strong evidence to this conclusion.

Arg-1 is considered a crucial marker of M2 macrophage polarization and catalyzes the hydrolysis of arginine to ornithine and urea, which are necessary for collagen production and fibrillation [8]. Arg-1 expression was significantly inhibited by LPS-induced M1 polarization, while A20 significantly promoted the expression of Arg-1. The expression of other M2 markers showed the same trend. As pleiotropic cytokines, IL-4 and IL-10 can inhibit the secretion of proinflammatory factors and play an important role in immune regulation. Transforming growth factor-β1 belongs to the TGF-β superfamily and regulates cell growth and differentiation. TGF-β1 was reported to have potential application prospects in treating wound healing and promoting cartilage and bone repair [33]. Our results showed that the expression of TGF-β1 was significantly upregulated in the A20 group, which indicated that A20 might have greater advantages in wound healing than the random nanofibers.

The expression of proinflammatory factors such as iNOS, TNF-α and IL-1β increased significantly in the M1 phenotype, but this effect was suppressed in the A20 group. TNF-α is the earliest and most important inflammatory mediator in the inflammatory response. IL-1β plays a key role in multiple inflammatory diseases. iNOS is considered a marker of M1 macrophages. iNOS is activated by inflammatory cytokine transcription, which leads to increased levels of nitric oxide (NO) during inflammatory responses [6, 34]. In general, macrophages in the A20 group showed a trend toward M2-type polarization in the presence of aligned fibers, which could reduce the expression of inflammatory factors and relieve inflammation. In addition, A20 promoted the expression of anti-inflammatory factors, which had a positive effect on later tissue repair.

### Aligned nanofibers suppressed M1 polarization via the JAK-STAT and NF-κB signaling pathways

**Fig. 3:**
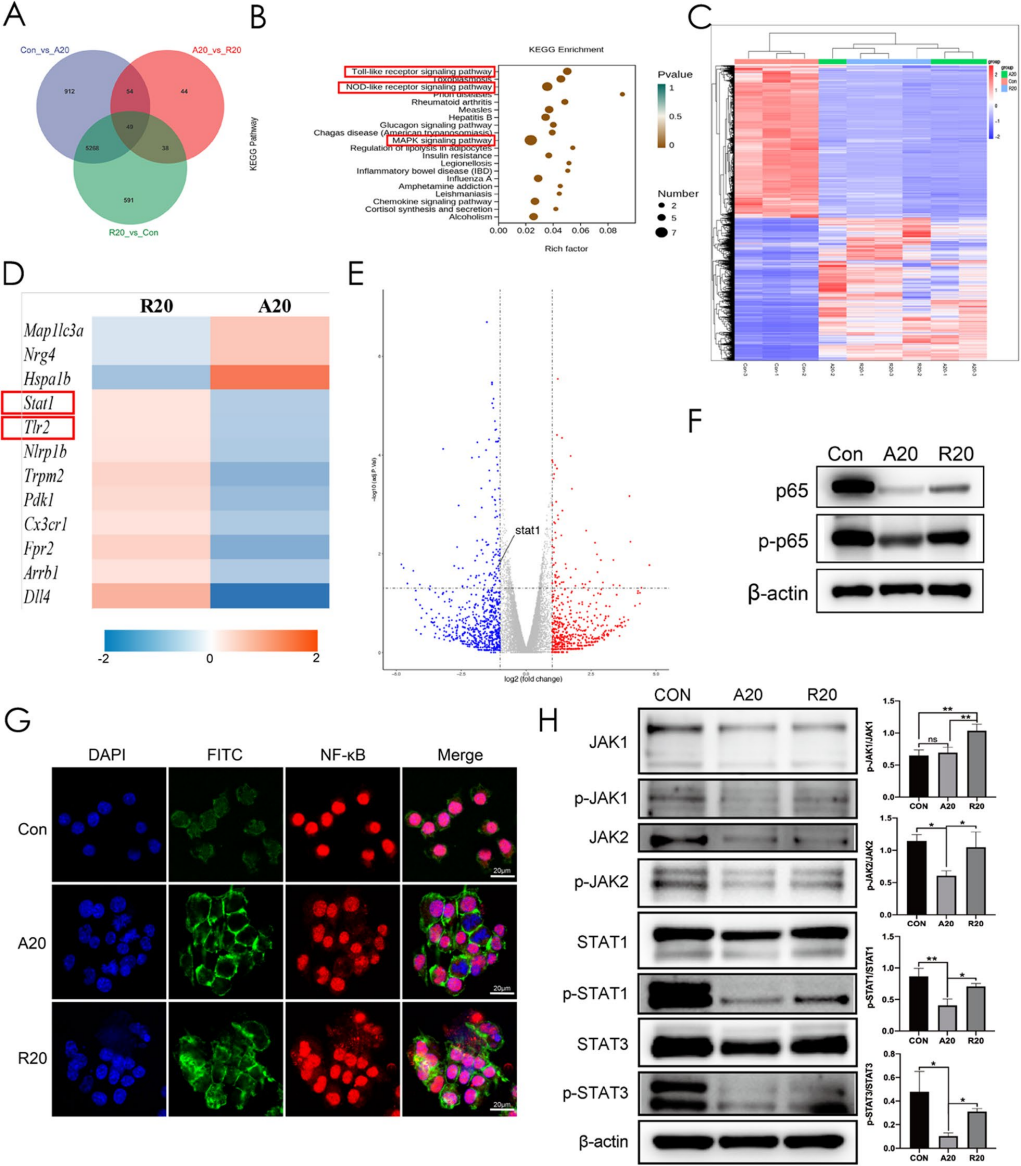
The underlying mechanism by which aligned fibers affected macrophage polarization. **A** Venn diagram showing differentially expressed genes. **B** KEGG pathway analysis between the A20 and R20 groups. **C** Heatmap of differentially expressed genes among the three groups. **D** Heatmap of macrophage polarization-related genes between the A20 and R20 groups. **E** Volcano diagram of differentially expressed genes. **F** Western blot analysis of the NF-κB signaling pathway. **G** Immunofluorescence staining showing the nuclear translocation of NF-κB p65. The nucleus is stained blue, and NF-κB p65 protein is stained red. **H** Western blot images and semiquantitative analysis of the JAK-STAT signaling pathway (*p < 0.05, **p < 0.01, n = 3)

To further examine the mechanism, mRNA-seq was performed. As shown in Fig. 3A, there were 49 differentially expressed genes among the three groups. The heatmap of differentially expressed genes among the three groups is shown in Fig. 3C. When both experimental groups were compared with the Con group, the differentially expressed genes in the A20 and R20 groups were consistently upregulated or downregulated compared with those in the Con group. Therefore, only changes in gene expression between the A20 and R20 groups were subsequently analyzed. KEGG signaling pathway analysis showed that the TLR, NOD-like receptor and MAPK pathways correlated with the effects of A20 on macrophage polarization (Fig. 3B). The heatmap in Fig. 3D indicates that the expression of M1 phenotype polarization-related genes, such as STAT1 and TLR2, was significantly downregulated in the A20 group. A volcano diagram shows the same trend for STAT1 (Fig. 3E).

To further validate the RNA-seq results, Western blot analysis was performed. The results showed downregulated expression of p-NF-κB p65 in the A20 group (Fig. 3F). Immunofluorescence staining showed that the nuclear translocation of NF-κB P65 in the A20 group was significantly lower than that in the other groups (Fig. 3G). In addition, the JAK-STAT signaling pathway (p-JAK1, p-JAK2, p-STAT1 and p-STAT3) was inhibited by A20 treatment. Semiquantitative analysis showed that the difference was statistically significant (Fig. 3H).

The RNA-seq results showed that the MAPK, NOD-like receptor and TLR signaling pathways were highly associated with the difference between the A20 and R20 groups. For example, TLR2 was downregulated in the A20 group, as shown on the heatmap. It has been widely documented that the NF-κB signaling pathway, as the downstream of the TLR signaling pathway, plays an important role in macrophage polarization. In response to intracellular stimulation, such as LPS and proinflammatory cytokines, NF-κB translocates to the nucleus. In the nucleus, NF-κB binds to target genes to facilitate transcription [35]. Thus, nuclear translocation is a key part of NF-κB signaling pathway activation. The immunofluorescence staining results verified the lowest colocalization of NF-κB p65 with the nucleus in the A20 group, indicating that A20 inhibited NF-κB pathway activation.

The JAK-STAT signaling pathway is involved in numerous vital biological processes, such as cell proliferation, differentiation, apoptosis, and immune regulation. When cytokines bind to the receptor, JAK is phosphorylated and activated, which subsequently leads to phosphorylation and dimerization of STAT [36, 37]. The activated STAT dimer migrates to the nucleus and binds to specific DNA sites, ultimately causing changes in cell function. STAT1, which is a major transcription factor that is activated by IFN, plays a crucial role in normal immune responses and is critical for M1 macrophage polarization. STAT1 activates the expression of several proinflammatory genes. It has been reported that suppressing STAT1 facilitates M2 macrophage polarization [38].

Both NF-κB and JAK-STAT are involved in regulating cell behaviors. NF-κB and JAK-STAT signaling pathways can independently cause cytokine storms that regulate immune inflammatory responses, while the secretion of inflammatory factors can in turn initiate each other’s responses. According to previous reports, STAT-NF-κB synergistically shaped the transcriptional response to infection [39]. And the synergistic effects between the NF-κB and STAT1 pathways might be the main pathway of M1 polarization [6]. All these studies verified that there is certain interaction between NF-κB and JAK-STAT pathways. Our RNA-seq and Western blot results indicated that the inflammatory process was suppressed via the NF-κB and JAK-STAT signaling pathways in the A20 group. However, this modulatory effect might be achieved through the indirect interactions between NF-κB and JAK-STAT pathways rather than the direct interactions of protein-colocalization.

### Aligned nanofibers facilitated wound healing in the skin defects in mice

**Fig. 4:**
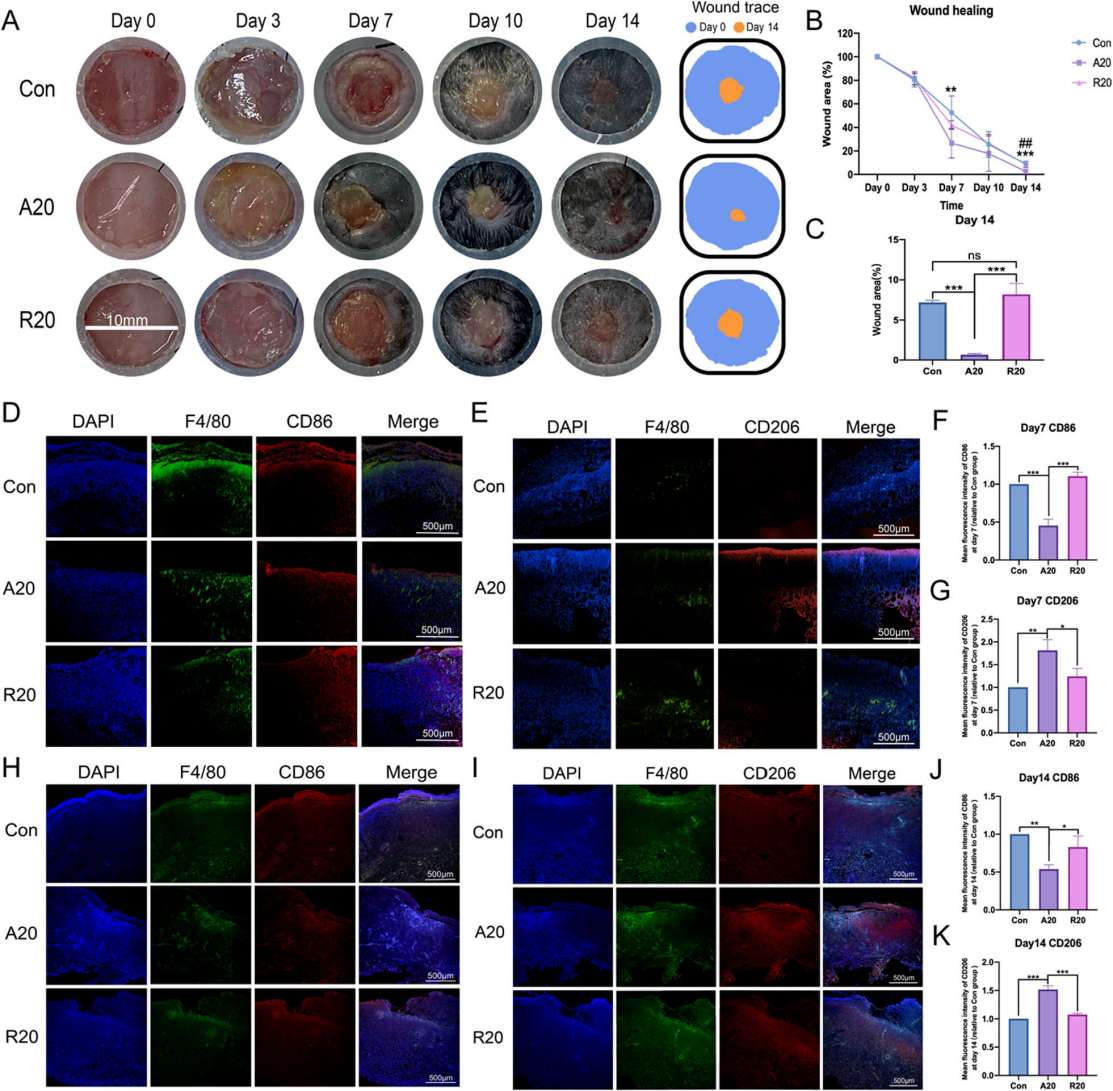
The effect of electrospun membranes on wound healing in skin defects in mice. **A** Skin defect healing in mice at different time points. **B** Line diagram showing skin defect healing in mice at different time points. * indicates a significant difference between the A20 and Con groups, ^#^ indicates a significant difference between the A20 and R20 groups. **C** Statistical analysis of the remaining wound area in the three groups on day 14. (**p < 0.01, ^##^*p* < 0.01, ***p < 0.001, n = 5). **D**, **E** Immunofluorescence staining of CD86 and CD206 in each group of skin sections on day 7. **F**, **G** Mean fluorescence intensity of CD86 and CD206 in each group of skin sections on day 7. **H**, **I** Immunofluorescence staining of CD86 and CD206 in each group of skin sections on day 14. **J**, **K** Mean fluorescence intensity of CD86 and CD206 in each group of skin sections on day 14. DAPI (blue) was used to label the nucleus, F4/80 (green) was used to label macrophages, and CD86 or CD206 (red) was used to label M1- or M2-phenotype macrophages, respectively (*p < 0.05, **p < 0.01, *** *p* < 0.001, n = 3)

The dorsal skin defects of mice in the three groups healed over time, and wound healing was significantly promoted in the A20 group. The residual wound area in the A20 group was the smallest on day 14, and the wound trace diagram made the results more intuitive (Fig. 4A). The line chart showed a similar trend, and there was a significant difference between the A20 group and the Con group on day 7 and a significant difference between the A20 group and the other two groups on day 14 (Fig. 4B and C).

Immunofluorescence staining was performed to observe the distribution of M1 and M2 macrophages in skin tissue sections on days 7 and 14. Immunofluorescence images on day 7 showed a large number of CD86-labeled M1 macrophages in all three groups; however, a relatively lower number of M1 macrophages was observed in the A20 group (Fig. 4D). Immunofluorescence staining for iNOS at the two time points also showed similar results (Additional file 1: Fig. S2). CD206-positive cells were significantly increased in the A20 group compared to the other two groups and were mainly concentrated in the sites of the skin defects (Fig. 4E). On day 14, the A20 group still exhibited the fewest CD86^+^ cells (Fig. 4H), and CD206^+^ cells were mostly found in the A20 group (Fig. 4I). The statistical analysis of the mean fluorescence intensity was shown in Fig. 4F, G, J and  K, and the differences were statistically significant.

Unlike humans, mice develop spontaneous contractures after skin defects [40]. Therefore, 10 mm diameter silicone was sewn onto the backs of the mice to alleviate contracture. Encouragingly, the A20 group showed accelerated healing results from day 7 onward. Hu et al. also reported similar results, and verified that aligned nanofibers could promote active extracellular matrix synthesis [41]. Based on our previous experiments, we hypothesized that A20 promoted the polarization of M2 macrophages in the wound area earliest and improved the anti-inflammatory response. Later, M2 macrophages produced a large number of cytokines to promote tissue regeneration, which could mobilize fibroblast differentiation and eventually promote wound healing. The immunofluorescence staining results also confirmed this hypothesis. Abundant M1 macrophages were observed in all three sections, indicating a progressive inflammation stage in wound sites. The high expression of CD206 in the A20 group suggested that vigorous tissue regeneration was taking place, which was also consistent with the progression of wound healing.

**Fig. 5:**
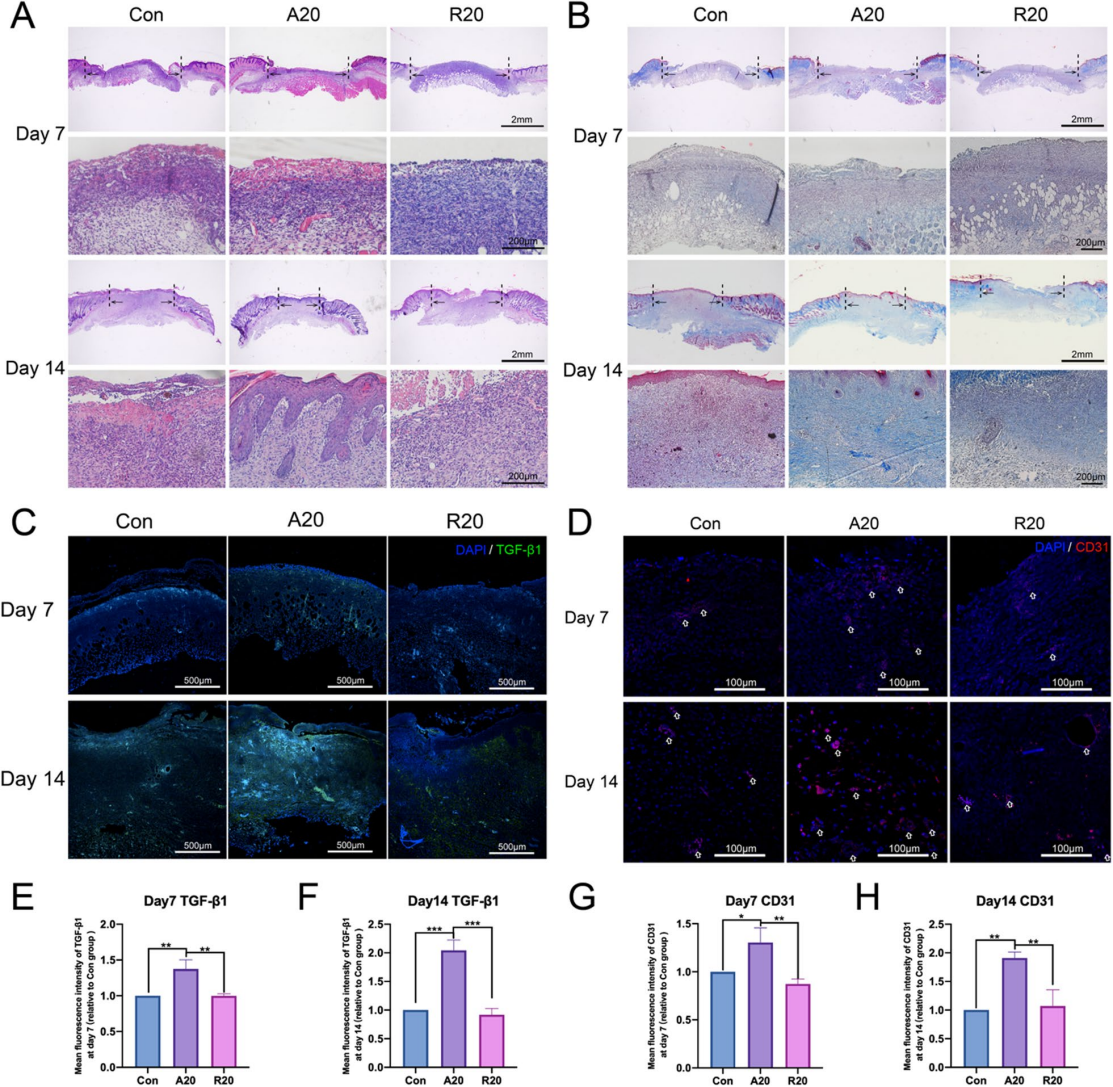
Histological analysis of skin defects in mice. **A** H&E staining in the three groups on days 7 and 14. The dotted lines indicate the size of the wound. **B** Masson staining in three groups on days 7 and 14. The dotted lines indicate the size of the wound. **C** Immunofluorescence staining of TGF-β1 in each group of skin sections on days 7 and 14. DAPI is stained blue, and TGF-β1 is stained green. **D** Immunofluorescence staining of CD31 in each group of skin sections on days 7 and 14. DAPI is stained blue, and CD31 is stained red. Arrows indicate the location of neovascularization. **E**, **F** Mean fluorescence intensity of TGF-β1 in each group of skin sections on days 7 and 14. **G**, **H** Mean fluorescence intensity of CD31 in each group of skin sections on days 7 and 14 (*p < 0.05, **p < 0.01, *** *p* < 0.001, n = 3)

Subsequently, HE and Masson staining was conducted to assess the process of wound healing. The H&E staining results are shown in Fig. 5A. The thickest regenerated granulation tissue and the fewest inflammatory cells, such as macrophages and neutrophils, were observed in the A20 group on day 7. In addition, there were more regenerated vessels in the A20 group than in the other groups. By the 14th day, a more complete epidermal layer was produced in the A20 group than in the other groups. The wound surface was covered with a more complete epidermal structure, more collagen deposition and fewer inflammatory cells were observed than in the other two groups. These results showed that the repair quality was the best in the A20 group.

Collagen deposition is an important indicator of wound healing. After Masson staining, the collagen fibers were stained blue, and their relative intensity indicated the collagen content [42]. On day 7, collagen was fully deposited and densely arranged in the A20 group, and the neoplastic epithelial layer was more visible than in the other groups. In addition, on day 14, collagen deposition was significantly higher in the A20 group than in the other groups, and collagen fibers were intertwined to form a network structure (Fig. 5B).

Furthermore, TGF-β1 and CD31 immunofluorescence staining was performed to investigate tissue regeneration and neovascularization in the skin samples. The immunofluorescence images of TGF-β1 (Fig. 5C) on days 7 and 14 showed that the level of TGF-β1 increased with time, and its expression was higher in the A20 group at both time points. Figure 5D shows the immunofluorescence staining of CD31, and that there was more CD31-labeled neovascularization in the A20 group on days 7 and 14. The statistical analysis of the mean fluorescence intensity of TGF-β1 and CD31 on days 7 and 14 were shown in Fig. 5E, F, G and H, and the differences were statistically significant.

Tissue-resident macrophages may be the earliest responders to traumatic injury. After injury, the cells synthesize adhesion molecules that recruit and direct a variety of cell types. These macrophages are subsequently highly polarized to the M2 phenotype in response to IL-4 and coordinate the wound healing phase [43]. M2 macrophages can express TGF-β1, a multifunctional growth factor that regulates the proliferation, migration and differentiation of functional cells, modulates ECM production, exerts immunomodulatory effects, and ultimately promotes wound healing.

The proliferation stage of wound healing mainly depends on fibroblasts, which are responsible for initiating angiogenesis and synthesizing collagen fibers [44]. We hypothesized that aligned nanofibers polarized macrophages to the M2 phenotype, which contributed to the migration and proliferation of fibroblasts. In addition, TGF-β1 secreted by M2 macrophages promoted not only the proliferation of fibroblasts and epidermal cells but also the synthesis of extracellular matrix, which was closely related to wound healing and affected almost every stage of wound healing [45]. In a model of idiopathic pulmonary fibrosis, the production and activation of TGF-β1 was responsible for the profibrotic effects of macrophages [46]. In addition, a robust and dynamic angiogenic response has been reported to be essential for wound healing, and new capillaries provide nutrients and oxygen for wound healing [47, 48]. Macrophages can mediate angiogenesis in wound healing and secrete regulatory factors to influence neointimal growth [49]. A widely accepted opinion is that the M2 phenotype is closely associated with angiogenic activity through the synthesis of angiogenic growth factors [50, 51].

### Macrophage-conditioned medium from the A20 group promoted the biological behaviors of L929 fibroblasts and MAECs

**Fig. 6:**
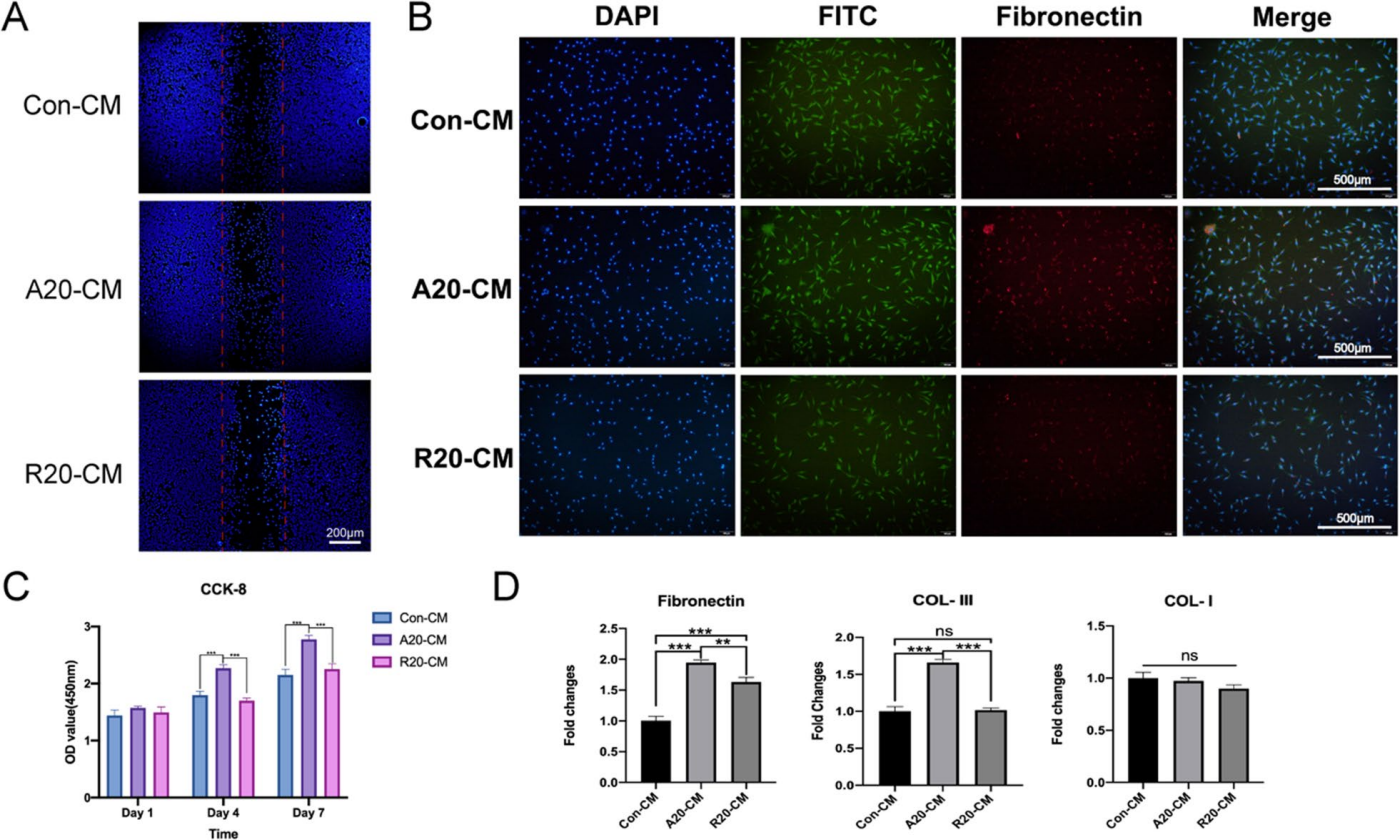
The effect of macrophage-conditioned medium on the biological behaviors of L929 fibroblasts. **A** Migration of L929 cells. **B** Immunofluorescence staining of fibronectin. Actin is stained green, nuclei are stained blue, and fibronectin is stained red. **C** CCK-8 analysis of the proliferation of L929 cells. **D** qPCR analysis of collagen formation-related genes. (*p < 0.05, **p < 0.01, ***p < 0.001, n = 3)

Next, the effect of macrophage conditioned medium on the biological behaviors of L929 and MAEC cells was explored. As shown in Fig. 6A, A20-CM accelerated the migration of L929 fibroblasts compared with that in the Con-CM and R20-CM groups. Immunofluorescence staining showed that the A20-CM group expressed higher levels of fibronectin than the Con-CM and R20-CM groups (Fig. 6B). The CCK-8 assay showed that the OD value in the A20-CM group was significantly higher than that in the other groups on days 4 and 7 (Fig. 6C). Moreover, qPCR was used to measure the expression of collagen formation-related genes, and A20-CM significantly upregulated the expression of fibronectin and COL-III in L929 fibroblasts; COL-I was not significantly different among the three groups (Fig. 6D).

The CCK-8 assay results (Additional file 1: Fig. S3) indicated that all the conditioned medium promoted the proliferation of MAECs, and the OD value in the A20-CM group was significantly higher than that in the other groups on day 7. Additional file 1: Fig. S4 shows that A20-CM facilitated the migration of MAECs. The results of qPCR (Additional file 1: Fig. S5) showed that A20-CM upregulated the expression of angiogenesis related genes.

Fibroblasts play an important role in skin wound healing by promoting angiogenesis and facilitating epithelialization and collagen production. After injury occurs, fibroblasts migrate to the damaged sites and proliferate under the stimulation of cytokines secreted by M2 macrophages, which is the basis for tissue reconstruction. Angiogenesis is also essential for the maintenance of granulation tissue and is associated with the activity of a large number of cytokines (e.g., bFGF and TGF-β). Granulation tissue formation, collagen deposition and angiogenesis occur simultaneously with epithelialization and wound contraction. Subsequently, fibroblasts secrete type III collagen and fibronectin, resulting in mechanically strong tissue. Fibronectin is essential in all three phases of wound healing: inflammation, proliferation, and remodeling [52]. Fibronectin regulates cell adhesion kinetics and subsequently provides the necessary ECM templates for collagen deposition [53]. A20-CM promoted the migration and proliferation of fibroblasts in vitro and upregulated the expression of fibronectin and COL-III. According to our previous experiments, we hypothesize that macrophages cultured on aligned nanofibers synthesized increased levels of regenerative factors, such as TGF-β1, which could result in higher expression of fibronectin and COL-III. Relevant studies also indicated that TGF-β1 could induce cellular expression of fibronectin in skin wounds to promote repair and wound healing [54].

## Conclusions

In this study, we fabricated aligned and random PLLA nanofibers. Improved mechanical characteristics and hydrophilia were observed in the A20 group. In vitro, highly aligned fibrous structures in the A20 group induced macrophages to polarize toward the M2 phenotype. The A20 group had significantly attenuated expression of proinflammatory genes and promoted anti-inflammatory gene expression. Most importantly, our results revealed that A20 inhibited macrophage M1 polarization via the JAK-STAT and NF-κB signaling pathways. In addition, we verified that A20-CM significantly facilitated L929 fibroblast migration, proliferation, differentiation and fibronectin expression. In vivo, A20 accelerated wound healing in the skin defects of mice, attenuated inflammation, and promoted epidermal regeneration, collagen fiber deposition and neovascularization. Overall, our study demonstrates that well-aligned electrospun nanofibers can suppress the M1 macrophage phenotype and inhibit inflammatory progression via the JAK-STAT and NF-κB signaling pathways and thus hold promise as an ideal dressing for wound healing.

## Supplementary Information

**Additional file 1: Table S1.** Gene primer sequences for q-PCR. **Fig. S1.** Diameter distribution of electrospun fibers of A20 and R20 groups. **Fig. S2.** (A & B) Immunofluorescence staining of iNOS at days 7 and 14. (C & D) Mean fluorescence intensity of iNOS in each group of skin sections on days 7 and 14. Nucleus was stained blue and iNOS was stained red. Scale bar = 200 μm. **Fig. S3. ** CCK-8 assay of MAEC culturing with macrophage conditioned medium. (*p < 0.05, n=3). **Fig. S4.** Migration of MAEC under conditional medium. Scale bar = 500 μm. **Fig. S5. **The expression of angiogenesis related genes of MAEC cultured with macrophage conditioned medium. (*p < 0.05, **p < 0.01, n = 3)
