# Supplementary material for: Aligned electrospun poly(l-lactide) nanofibers facilitate wound healing by inhibiting macrophage M1 polarization via the JAK-STAT and NF-κB pathways
Source: J Nanobiotechnology. 2022 Jul 26;20:342. doi: 10.1186/s12951-022-01549-9 (PMC9327399; doi:10.1186/s12951-022-01549-9)
Supplement: Supplementary file 1 — Additional file 1: Table S1. Gene primer sequences for q-PCR. Fig. S1. Diameter distribution of electrospun fibers of A20 and R20 groups. Fig. S2. (A & B) Immunofluorescence staining of iNOS at days 7 and 14. (C & D) Mean fluorescence intensity of iNOS in each group of skin sections on days 7 and 14. Nucleus was stained blue and iNOS was stained red. Scale bar = 200 μm. Fig. S3. CCK-8 assay of MAEC culturing with macrophage conditioned medium. (*p < 0.05, n=3). Fig. S4. Migration of MAEC under conditional medium. Scale bar = 500 μm. Fig. S5. The expression of angiogenesis related genes of MAEC cultured with macrophage conditioned medium. (*p < 0.05, **p < 0.01, n = 3) [file 12951_2022_1549_MOESM1_ESM.docx]

Supplementary Information

**Aligned Electrospun Poly(L-lactide) Nanofibers Facilitate Wound Healing by Inhibiting Macrophage M1 Polarization via JAK-STAT and NF-κB Pathways**

Jian Xie ^1^, Xiaowei Wu ^2^, Shang Zheng ^1^, Kaili Lin ^3,*^, and Jiansheng Su ^1,*^

^1^ Department of Prosthodontics, Stomatological Hospital and Dental School of Tongji University, Shanghai Engineering Research Center of Tooth Restoration and Regeneration, Shanghai 200072, China

^2^ Department of orthodontics, Shanghai Ninth People’s Hospital, College of Stomatology, Shanghai Jiao Tong University School of Medicine; National Clinical Research Center for Oral Diseases; Shanghai Key Laboratory of Stomatology & Shanghai Research Institute of Stomatology, Shanghai 200125, China

^3^ Department of Oral & Cranio-Maxillofacial Surgery, Shanghai Ninth People’s Hospital, College of Stomatology, Shanghai Jiao Tong University School of Medicine; National Clinical Research Center for Oral Diseases; Shanghai Key Laboratory of Stomatology, Shanghai Research Institute of Stomatology, Shanghai 200125, China

**^*^ Corresponding authors**

E-mail adress: linkaili@sjtu.edu.cn & [lklecnu@aliyun.com](mailto:lklecnu@aliyun.com) (K. Lin), [sjs@tongji.edu.cn](mailto:sjs@tongji.edu.cn) (J. Su)

**Table S1**. Gene primer sequences for q-PCR.

| Gene | | Forward primer (5' - 3') | Reverse primer (5' - 3') | |
| --- | --- | --- | --- | --- |
| IL-1β  TNF-α  iNOS  Arg-1  IL-4  IL-10  TGF-β1  GAPDH  Fibronectin  COL-III  COL-I  b-FGF  eNOS  KDR | GAAATGCCACCTTTTGACAGTG  CCTGTAGCCCACGTCGTAG  GTTCTCAGCCCAACAATACAAGA  CTCCAAGCCAAAGTCCTTAGAG  GGTCTCAACCCCCAGCTAGT  TACAGCCGGGAAGACAATAA  GTCCTTGCCCTCTACAACCA  AGGTCGGTGTGAACGGATTTG  CGAGGTGACAGAGACCACAA  CTGTAACATGGAAACTGGGGAAA  GCTCCTCTTAGGGGCCACT  GGAGAAGAGCGACCCACACG  GGATTCTGGCAAGACCGATTACA  TCTGTGGTTCTGCGTGGAGA | | | TGGATGCTCTCATCAGGACAG  GGGAGTAGACAAGGTACAACCC  GTGGACGGGTCGATGTCAC  AGGAGCTGTCATTAGGGACATC  GCCGATGATCTCTCTCAAGTGAT  AAGGAGTCGGTTAGCAGTAT  GTTGGACAACTGCTCCACCT  GGGGTCGTTGATGGCAACA  CTGGAGTCAAGCCAGACACA  CCATAGCTGAACTGAAAACCACC  CCACGTCTCACCATTGGGG  GCAGACATTGGAAGAAACAG  ACTTGTCCAAACACTCCACGCTG  GTATCATTTCCAACCACCCT |


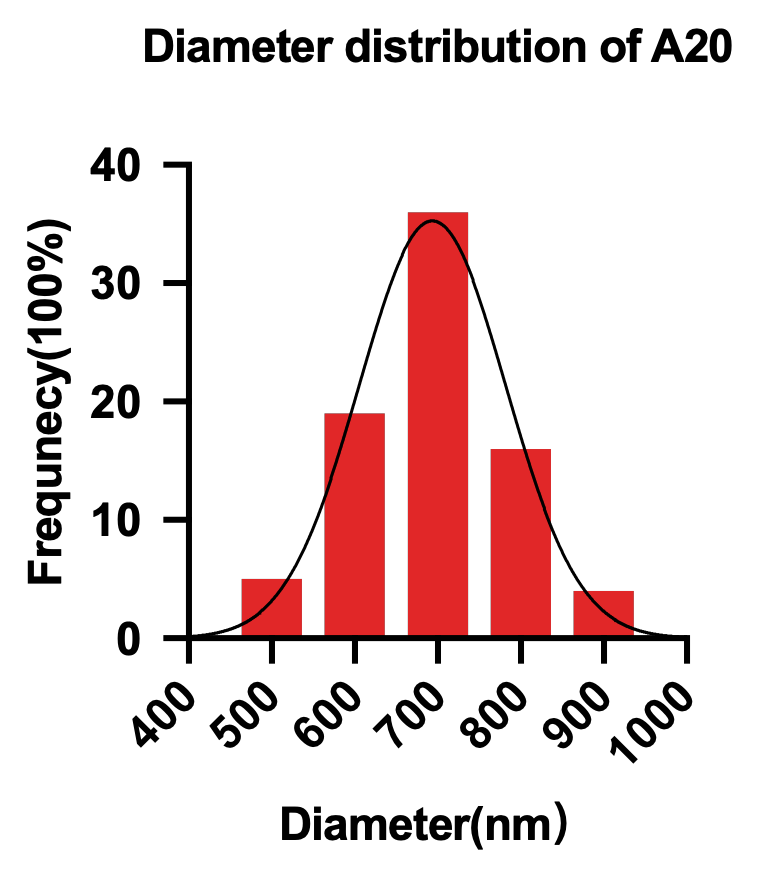

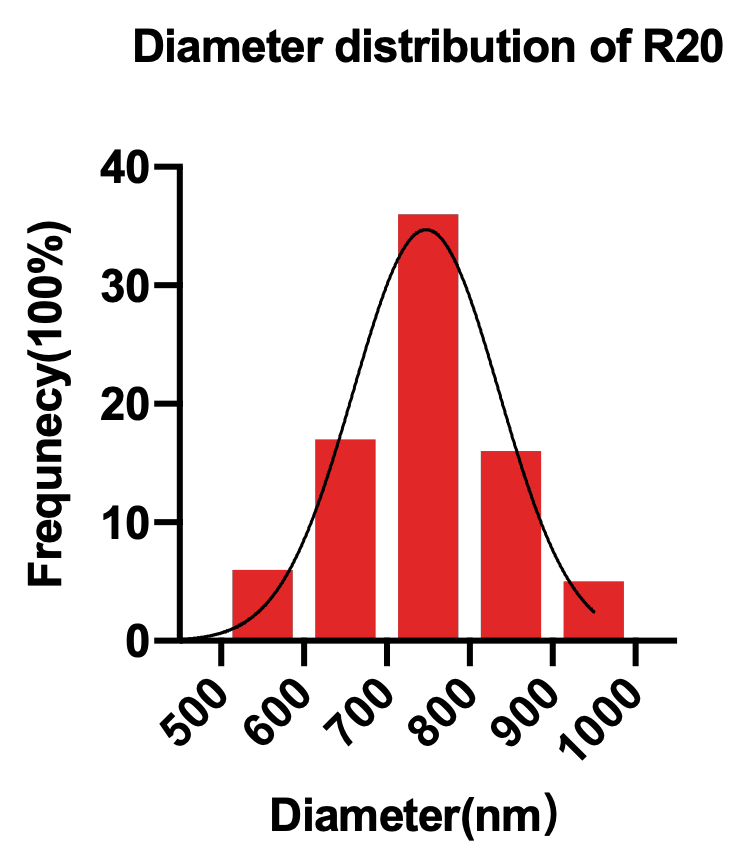


**Fig. S1**. Diameter distribution of electrospun fibers of A20 and R20 group.


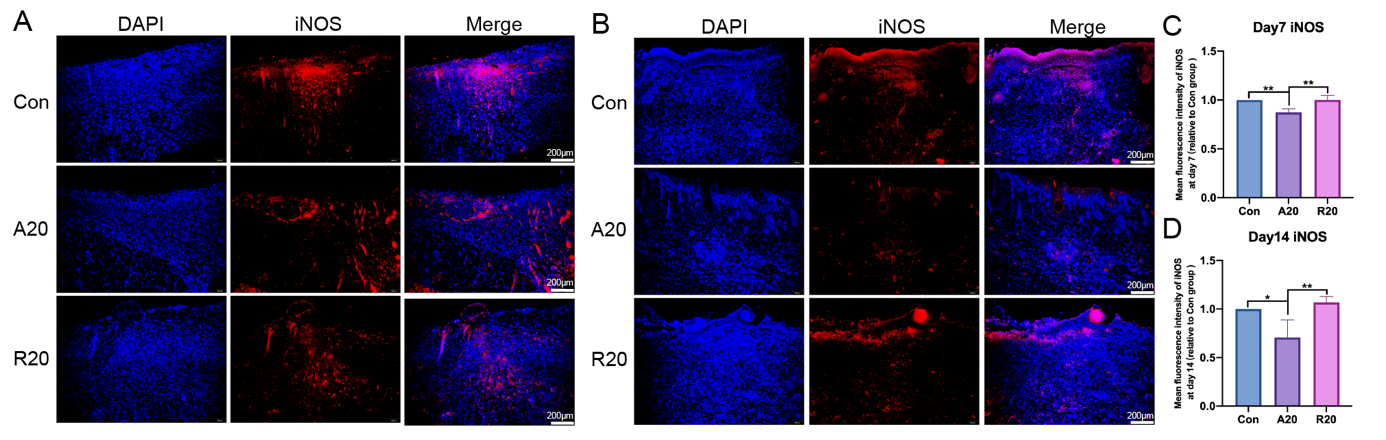


**Fig. S2**. (A & B) Immunofluorescence staining of iNOS at days 7 and 14. (C & D) Mean fluorescence intensity of iNOS in each group of skin sections on days 7 and 14. Nucleus was stained blue and iNOS was stained red. Scale bar = 200 μm.


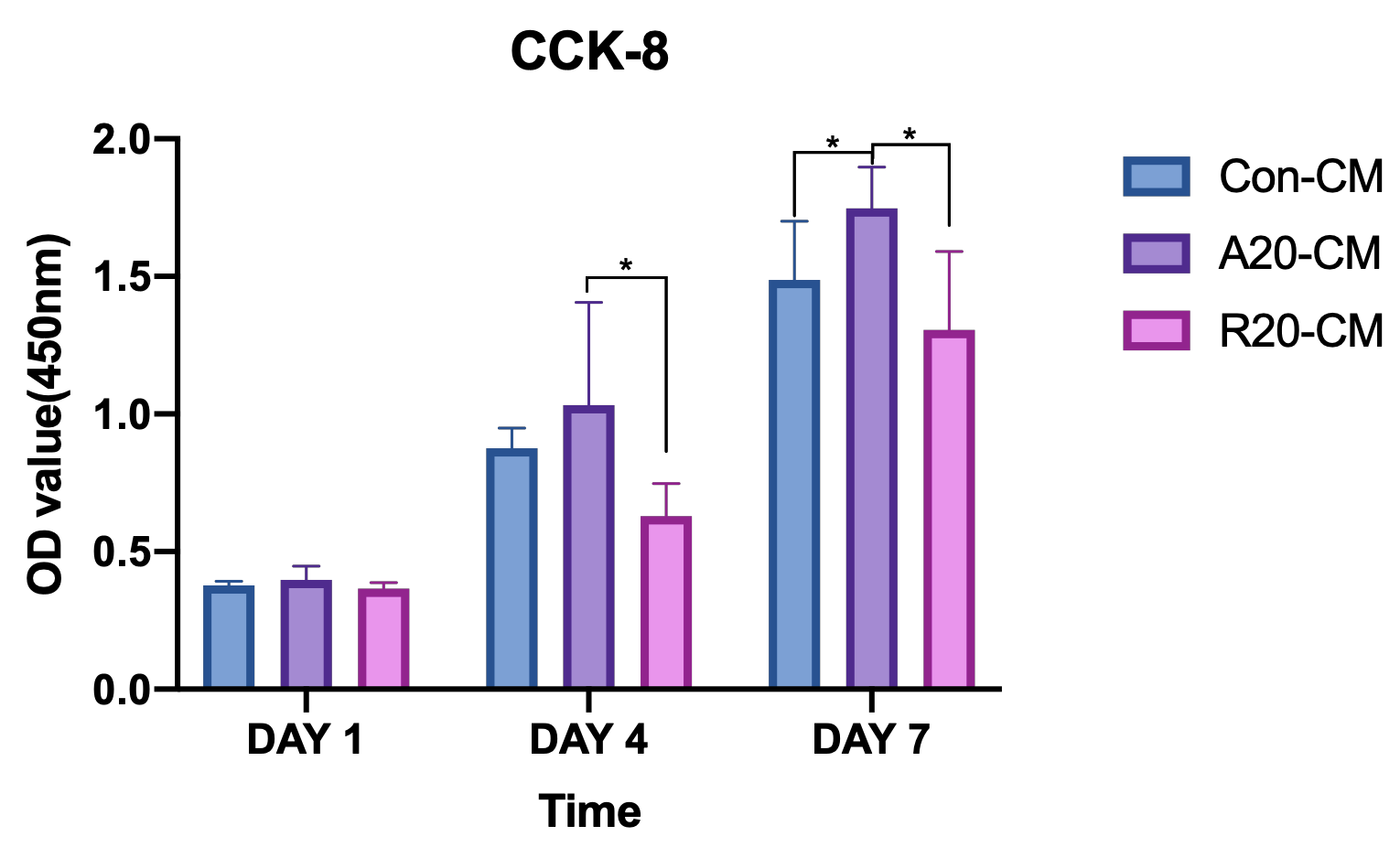


**Fig. S3**. CCK-8 assay of MAEC culturing with macrophage conditioned medium. (**p* < 0.05, n=3)


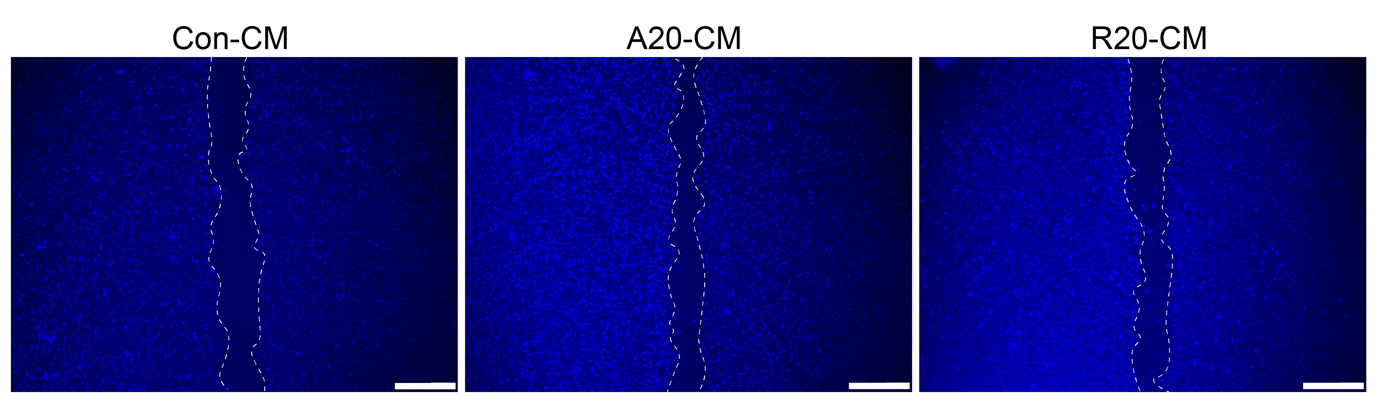


**Fig. S4**. Migration of MAEC under conditional medium. Scale bar = 500 μm.


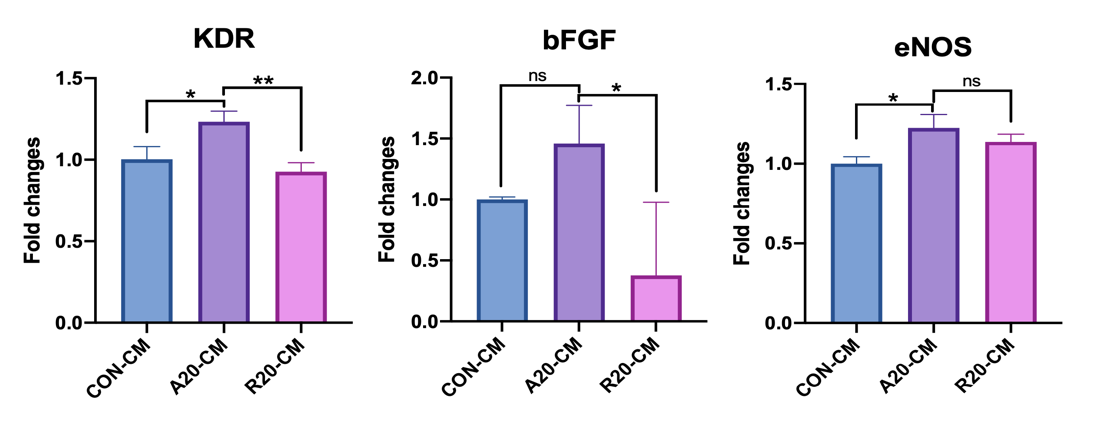


**Fig. S5**. The expression of angiogenesis related genes of MAEC cultured with macrophage conditioned medium. (**p* < 0.05, ***p* < 0.01, n = 3)
